# Blood Plasma Stabilized Gold Nanoclusters for Personalized Tumor Theranostics

**DOI:** 10.3390/cancers14081887

**Published:** 2022-04-08

**Authors:** Greta Jarockyte, Vilius Poderys, Virginijus Barzda, Vitalijus Karabanovas, Ricardas Rotomskis

**Affiliations:** 1 Biomedical Physics Laboratory, National Cancer Institute, LT-08406 Vilnius, Lithuania; greta.jarockyte@nvi.lt (G.J.); vilius.poderys@nvi.lt (V.P.); ricardas.rotomskis@nvi.lt (R.R.); 2 Life Science Center, Vilnius University, LT-10257 Vilnius, Lithuania; 3 Laser Research Center, Faculty of Physics, Vilnius University, LT-10223 Vilnius, Lithuania; virgis.barzda@utoronto.ca; 4 Department of Chemical and Physical Sciences, University of Toronto Mississauga, Toronto, ON L5L 1C6, Canada; 5 Department of Physics, University of Toronto, Toronto, ON M5S 1A7, Canada; 6 Department of Chemistry and Bioengineering, Vilnius Gediminas Technical University, LT-10223 Vilnius, Lithuania

**Keywords:** biosynthesis, nanomedicine, fluorescent diagnostic, photodynamic cancer therapy

## Abstract

**Simple Summary:**

Cancer is a disease that has a high fatality rate over the world. Nanotechnology is one of the most promising current approaches for developing novel diagnostic and treatment methods in accomplishing more personalized medicine. Personalized gold nanoclusters have potential to be used in cancer theranostics. We demonstrate that biocompatible gold nanoclusters could be synthesized directly in human blood plasma. Such gold nanoclusters have a wide photoluminescence band in the optical tissue window and generate reactive oxygen species under irradiation with visible light, thus are suitable for cancer theranostics.

**Abstract:**

Personalized cancer theranostics has a potential to increase efficiency of early cancer diagnostics and treatment, and to reduce negative side-effects. Protein-stabilized gold nanoclusters may serve as theranostic agents. To make gold nanoclusters personalized and highly biocompatible, the clusters were stabilized with human plasma proteins. Optical properties of synthesized nanoclusters were investigated spectroscopically, and possible biomedical application was evaluated using standard cell biology methods. The spectroscopic investigations of human plasma proteins stabilized gold nanoclusters revealed that a wide photoluminescence band in the optical tissue window is suitable for cancer diagnostics. High-capacity generation of singlet oxygen and other reactive oxygen species was also observed. Furthermore, the cluster accumulation in cancer cells and the photodynamic effect were evaluated. The results demonstrate that plasma proteins stabilized gold nanoclusters that accumulate in breast cancer cells and are non-toxic in the dark, while appear phototoxic under irradiation with visible light. The results positively confirm the utility of plasma protein stabilized gold nanoclusters for the use in cancer diagnostics and treatment.

## 1. Introduction

Cancer remains one of the diseases with a high level of complexity that necessitates multi-step diagnostic and treatment procedures. With rapid nanotechnology development, there has been, recently, an increased use of nanomedicine in oncology. In the last decade, ample efforts were dedicated to the development of molecular and particle agents that can be used for both cancer diagnosis and treatment. The field of such research known as “theranostics”, a term used to characterize the combined therapeutic and diagnostic tasks performed by a single system, has resulted in a more precise cancer diagnosis and therapy. These multimodal theranostic platforms combine the diagnostics and treatment, and, therefore, can eliminate multi-step medical procedures, reduce delays in treatment, and improve patient-care and early diagnostics. As a result, there is a close relationship between diagnostics and therapies, which can lead to a more tailored treatment with a better prognosis. Personalization of such theranostic platforms would allow to achieve better outcome, because of consideration of specific patient condition, which would lead to reduced side effects. Personalized cancer theranostics offers various advantages, including improved diagnostics, tumor specific delivery of drugs, reduced local toxicity and lethal effects for normal tissues.

One of main directions of cancer theranostics is the combination of optical biopsy methods and photodynamic tumor therapy (PDT). Traditional photosensitizers (PS), such as hematoporphyrin or chlorin e_6_ (Ce_6_), could be considered as primary theranostic materials because they exhibit both non-invasive optical diagnostics and phototherapeutic activity. However, porphyrin-type PS have some drawbacks, such as lack of accumulation specificity, low solubility in water, low photostability, as well as cytotoxicity in the dark. Although most of drawbacks could be overcome by chemical engineering, all attempts to improve accumulation specificity were not successful. After injection, PS tend to accumulate not only in the tumor, but also in healthy tissues [1]. Accumulation of PS in skin makes the patient sensitive to light, which complicates the treatment. The progress in the development of nanoparticles (NPs) for medical applications prompted the use of various nanomaterials, such us quantum dots [2], upconverting nanoparticles [3], and gold nanoclusters (Au NCs) [4,5]. These NPs could be used as photo-drug targeted delivery vehicles; however, such bioconjugates often lack biocompatibility and colloidal stability. 

The simplicity, stability, and efficiency are crucial factors for any nanopharmaceutical translation from a laboratory to a clinic. The development of optically active NPs, which could produce a high amount of reactive oxygen species (ROS), allows to establish simplified nanoplatform for cancer theranostics. Au NCs are one of several possible nanoplatform which are investigated as promising PS for PDT, for example, glutathione protected Au NCs produce ^1^O_2_, which causes the death of cells under two-photon excitation using a pulsed 800 nm laser [6]. Recently, our group has also demonstrated the capability of bovine serum albumin (BSA) coated Au NCs (BSA–Au NCs) to generate reactive oxygen species upon light exposure and possible application of BSA–Au NCs for PDT in breast cancer cells [7]. In general, the application Au NCs for cancer diagnostics [8] and therapy has received ample attention [9] not only for PDT, but also for photothermal therapy [10,11], radiotherapy [12,13], and chemotherapy as the anti-cancer drug carrier [14,15].

Biocompatibility and organism response to NPs always were great concerns when considering NPs’ application for clinical therapy, and, thus, the search for non-toxic nanomaterials is important in further development of theranostic agents. Gold is one of the most biocompatible metals used for various medical devices and implants [16,17]; consequently gold NPs are expected to be non-toxic and suitable for theranostic bio-applications. A great deal of attention has been received for Au NCs as their size (<2 nm) and properties are similar to biomolecules. Amino and thiol groups bind to Au NCs; thus, surface modification with amino acids and proteins is also possible for those nanostructures. Since Xie et al. publication in 2009, when encapsulation of Au(III) ions with BSA was demonstrated [18], several other proteins, such us lysozyme [19,20], trypsin [21,22], ovalbumin [22], bovine [22,23] and human [24] insulin, human serum albumin (HSA) [25,26,27], as well as blood plasma proteins, such as fibrinogen [28] or transferrin [29,30], among others [31], have been also successfully used for Au NCs stabilization. Previously mentioned HSA is of particular interest because it is the most abundant protein in human plasma, that amounts to approximately 60% of all plasma proteins. The physiological concentration of albumin in blood plasma is 35–50 g/L, while concentrations of other proteins, such as immunoglobulins, fibrinogen, and transferrin, are much lower [32]. Current knowledge about Au NCs stabilization with individual plasma proteins opens the possibility for Au NCs biosynthesis directly in plasma. This approach will enable personalized cancer theranostics where Au NCs are synthesized directly in the patient’s plasma for increased Au NCs biocompatibility with the organism. So far, there have not been any reports about the Au NCs synthesis in human plasma. Currently, it is unclear if the formation of stabilized Au NCs would depend on blood type and Rhesus (Rh) factor.

Synthesis of protein stabilized Au NCs also is considered as green biosynthesis, because of eco-friendly and sustainable properties and no need for hazardous chemicals. These attributes allow biosynthesis of Au NCs in living organisms or media of biological origin. Wang et al. proved that Au NCs could be synthesized spontaneously by cancer cells [33]. Li et al. have demonstrated that biosynthesis of Au NCs is possible in the biological media, such as egg white and yolk, fetal bovine, and mouse and human serum [34]. In such a way, synthesized Au NCs have photoluminescence (PL) in the red region of 633–663 nm wavelength, when excited with 470 nm wavelength of light. The authors assumed that several different proteins stabilize Au NCs, as all used media has different sets of proteins, nevertheless, all synthesized Au NCs have similar pattern of PL spectra, such as BSA coated NCs, which may imply that albumins play the key role in Au NCs formation [18,34]. Later, a few publications described biosynthesis of Au NCs in bacteria, plant, and mushroom extracts [35,36]. 

The Au nanoclusters can be synthesized in a variety of biological media as mentioned above [18,19,20,21,22,23,24,25,26,27,28,29,30,31,34,35,36]. In this article, we focus on the personalized cancer therapy by using proteins of the human plasma. To our best knowledge, this is the first report on biosynthesis of Au NCs in human (or other mammalian) blood serum. Furthermore, we demonstrate a synthesis possibility of personalized Au nanoclusters for each individual that is fully compatible with the patient’s organism and causes the fewest side effects during theranostics. This approach will lead to a creation of fully personalized theranostic drugs.

The goal of this study is to investigate a possibility to synthesize Au NCs in human blood plasma and to study their photophysical and photosensitizing properties for possible applications in cancer theranostics. In our study, we demonstrate a direct synthesis of Au NCs in human blood plasma. We chose to use the two most universal groups of blood: group O Rh-positive (Rh+) and group O Rh-negative (Rh−). We assessed optimal conditions for Au NCs biosynthesis. It is shown that Au NCs stabilized with plasma proteins reveal a high efficacy of ROS generation under VIS irradiation and may act as a photo-drug in photodynamic therapy of cancer. Accumulation and biocompatibility of Au NCs are investigated in breast cancer cells. It leads to the conclusion that personalized Au NCs could be used in optical biopsy [37], for the cancerous tissue diagnostics and visualization of the border between cancerous and normal tissue. In addition, it should be stressed that Au NCs clusters stabilized by the human plasma could be used in tumor mammography, because the gold is a heavy metal which can be detected by using X-ray diagnostics methods [38,39]. Moreover, since gold nanoparticles (Au NPs) could be visualized by detecting third harmonic signals [40], we showed that Au NCs could be used as harmonophores for nonlinear microscopy as well. Finally, we evaluated the photodynamic effect of Au NCs in the cells. Our results demonstrate that Au NCs stabilized with human plasma proteins exhibit spectroscopic and photodynamic therapy properties that could be used for both cancer diagnostics and treatment.

## 2. Materials and Methods

### 2.1. Chemicals

HAuCl_4_ × aqua (~52% Au basis, M = 339.79 g/mol) and NaOH were purchased from Sigma-Aldrich (Darmstadt, Germany) and used without further purification. Deionized water was produced using ultrapure water system MicroPure UV (TKA, Dresden, Sachsen, Germany). Human plasma (blood group O (Rh+ and Rh−)) were acquired from Lithuanian national blood center. Before synthesis, human plasma was filtered using 0.22 µm syringe filters. The concentration of proteins in the plasma was determined by measuring absorption spectra of diluted plasma in the UV region and calculated using absorbance values at 280 nm and 260 nm. The following equation was used to calculate the mass concentration of proteins in plasma [41]:

(1) $$C_{protein}\left(\frac{\mathrm{mg}}{\mathrm{mL}}\right)=1.55\times A_{280}-0.76\times A_{260},$$

Dihydrorhodamine 123 (DHR123) (Invitrogen, USA) was chosen as a fluorescent probe to investigate the capacity of Au NCs Rh+/Rh− to generate reactive oxygen species when exposed to light. DHR123 is easily oxidized back to the parent fluorescent dye Rhodamine 123 (Rhod123) by ROS such as hydrogen peroxide [42,43]. Singlet Oxygen Sensor Green (SOSG) (Invitrogen, Waltham, MA, USA), a commercially available fluorescent sensor, was chosen as a highly sensitive probe for singlet oxygen with no noticeable reaction to hydroxyl radicals or superoxide [44,45]. SOSG is oxidized in the presence of ^1^O_2_, resulting in SOSG endoperoxides, which emit a bright green fluorescence with a maximum at 531 nm [46].

### 2.2. Au NCs Synthesis 

Synthesis procedure of plasma protein stabilized gold nanoclusters were performed using similar protocol of previously described synthesis of BSA–Au NCs [7,18,47]. Typically, NaOH solution (0.5 mL, 1.0 M) was added to human plasma (blood group O (Rh+/Rh−)) solution (5 mL, 37 °C, c = 50 mg/mL) under vigorous stirring. Later an aqueous HAuCl_4_ solution (5 mL, 37 °C, concentration depended on total protein/gold ratio) was slowly poured into the solution under vigorous stirring. The reaction was allowed to proceed under vigorous stirring for 12 h at the temperature of 37 °C. Different total protein: Au mass ratios (1 mg: 27 µg, 1 mg: 30 µg, 1 mg: 33 µg, 1 mg: 36 µg, 1 mg: 39 µg, 1 mg: 42 µg, 1 mg: 45 µg, 1 mg: 59 µg, 1 mg: 74 µg) were used for Au NCs Rh+/Rh− synthesis; however in both cases (Rh+ and Rh−) the most intense photoluminescence was observed using the above-mentioned synthesis procedure (total protein: Au mass ratio 1 mg: 36 µg). All further experiments were performed using these Au NCs solutions. 

Au NCs Rh+/Rh− solutions produced after the synthesis are alkaline (pH~11), and this could increase the cytotoxic effect for cells. To avoid this, PD MidiTrap G-25 columns (GE Healthcare, Chicago, IL, USA) were used for buffer exchange to PBS (pH = 7.2). Additionally, the solutions of Au NCs were filtered using a 0.02 µm syringe filter (polyethersulfone membrane, TPP, Switzerland) to prevent bacterial contamination in the cellular studies.

### 2.3. Characterization of Au NCs

The steady state absorption and photoluminescence spectra of synthesized Au NCs Rh+/Rh− were measured using UV-visible absorption spectrometer Varian Carry 50 (Varian Inc., Belrose, NSW, Australia) and fluorescence spectrometer Varian Cary Eclipse (Varian Inc.). Fluorescence decay measurements were performed using FLS 920 spectrometer (Edinburgh instruments Ltd., Livingston, UK) using the time correlated single photon counting technique. Diode laser (λ = 405 nm, pulse length—<200 ps, pulse repetition rate—100 kHz) was used for excitation of samples. Fluorescence decay was measured at the peak emission wavelength of the Au NCs Rh+/Rh− fluorescence band. All spectral measurements were performed in 1cm pathlength quartz cells (Hellma Optik GmbH, Jena, Germany).

A hydrodynamic diameter of particles was measured using the dynamic light scattering technique. Samples were measured using particle size and zeta potential analyzer Zeta Plus PALS (Brookhaven Inc., Suffolk County, NY, USA).

### 2.4. Generation of Reactive Oxygen Species

DHR123 (6 µL, 5 mM, M = 346.38 g/mol, Invitrogen, Waltham, MA, USA) was diluted with PBS (pH = 7.2) (Cegrogen-biotech, Ebsdorfergrund, Germany) to prepare a DHR123 stock solution (33 µM). Six types of samples were prepared for investigation of ROS generation. The first and second samples were made by diluting Au NCs Rh+/Rh− stock solutions (0.6 mL) with PBS (4.8 mL) and DHR123 stock solution (0.6 mL). The third sample was prepared by diluting a DHR123 stock solution (0.6 mL) with PBS (5.4 mL). The fourth and fifth samples were prepared by diluting the required amount of freshly filtered Rh+/Rh− plasma with PBS and DHR123 stock solution (0.6 mL). A sixth sample was prepared by diluting Ce_6_ with PBS and DHR123 stock solution (0.6 mL). The final volume of each prepared solution was 6 mL. The final concentration of proteins in prepared Au NCs Rh+/Rh− and Rh+/Rh− solutions was c = 5.5 mg/mL. Concentrations of DHR123 in all prepared solutions and concentration of Ce_6_ in the fifth solution were c = 3.30 × 10^−6^ M and c = 1.25 × 10^−6^ M, respectively.

SOSG (100 µg, Invitrogen, Waltham, MA, USA) was dissolved in methanol (33 µL), and then diluted with PBS (pH = 7.2) to prepare a SOSG stock solution (50 µM). Six types of samples were prepared for investigation of singlet oxygen generation. The first and second samples were made by diluting Au NCs Rh+/Rh− stock solutions (0.6 mL) with PBS (4.8 mL) and SOSG stock solution (0.6 mL). The third sample was prepared by diluting a SOSG stock solution (0.6 mL) with PBS (5.4 mL). The fourth and fifth samples were prepared by diluting the required amount of freshly filtered Rh+/Rh− plasma with PBS and SOSG stock solution (0.6 mL). The sixth sample was prepared by diluting Ce_6_ with PBS and SOSG stock solution (0.6 mL). The final volume of each prepared solution was 6 mL. The final concentration of protein in the prepared Au NCs Rh+/Rh− and Rh+/Rh− solutions was c = 5.5 mg/mL. Concentration of SOSG in all prepared solutions and concentration of Ce_6_ in the fifth solution were c = 5 × 10^−6^ M and c = 1.25 × 10^−6^ M, respectively.

The impact of applied light exposure on the samples was studied using a continuous wave diode laser (λ = 405 nm wavelength, I = 66 mW/cm^2^) (Roithner Lasertechnik GmbH, Germany). Each type of sample was split into two (each of 3 mL, for control and experiment), and poured into 1 cm polystyrene cuvettes, which were tightly sealed afterwards. The experiment samples were irradiated with 405 nm light under constant stirring, giving the total dose of 35.8 J/cm^2^. The steady state absorption and photoluminescence spectra of the Au NCs Rh+/Rh− and other solutions were measured using a UV-visible absorption spectrometer Carry 50 (Varian Inc.) and a fluorescence spectrometer Cary Eclipse (Varian Inc.). The spectra of both control and irradiated samples were measured after each period of irradiation.

### 2.5. Cell Culturing

As a model in vitro system for cellular experiments we chosen two human breast cancer cell lines MCF-7 and MDA-MB-231 (purchased from the European Collection of Cell Cultures and American Type Culture Collection, respectively). Cells were cultured in a cell growth medium (DMEM), supplemented with 10% (*v*/*v*) fetal bovine serum, 100 U/mL penicillin and 100 µg/mL streptomycin (all from Gibco, Thermo Fisher Scientific, Waltham, MA, USA). Cells were maintained at 37 °C in a humidified atmosphere containing 5% of CO_2_. The cells were routinely subcultured 2–3 times a week in 25 cm^2^ cells’ culture flasks. 

### 2.6. Biocompatibility Assay

MCF-7 and MDA-MB-231 cells were seeded on a 96-wellplate (TPP, Trasadingen, Switzerland) at a density of 1.5·10^3^ cells/well. After 24 h, the old medium was replaced with a fresh medium containing 2.75, 5.5, 13.75 or 27.5 mg/mL Au NCs Rh+/Rh−, while media alone without Au NCs was a control. Cells were incubated for 24 h in the dark. The next day Pierce LDH Cytotoxicity Assay Kit (Pierce Biotechnology, Thermo Scientific, USA) was used to detect extracellular appearance of lactate dehydrogenase (LDH). The concentration of extracellular LDH was quantified by measuring absorbance at 490 and 630 nm wavelength with plate-reading spectrophotometer (BioTek, Winooski, VT, USA). After obtaining absorbance values, they were recalculated as percentage values of cytotoxicity, according to the protocol. For better data representation estimated cytotoxicity (%) was recalculated to viability of cells (100 %—cytotoxicity (%)). Data are expressed as mean ± standard deviation (SD). The statistical significance of differences between studied groups was assessed using a two-tailed independent Student’s t-test at the 95% confidence level. Significance was represented as *p*-value < 0.05.

### 2.7. Accumulation of Au NCs in Cell Monolayers 

For intracellular imaging studies, MCF-7 and MDA-MB-231 cells were seeded into an 8-chambered cover glass plate (Thermo Fisher, USA) with a density of 3·10^4^ cells/chamber. For the evaluation of Au NCs uptake and intracellular localization, cells were treated with 13.75 mg/mL of Au NCs and incubated for the next 24 h. Nuclei of the cells were stained with 0.01 mg/mL Hoechst 33258 (Sigma-Aldrich, Steinheim am Albuch, Baden-Württemberg, Germany).

The accumulation of Au NCs was observed using a Nikon Eclipse Te2000-S C1 Plus laser scanning confocal microscope (Nikon, Minato-ku, Japan) equipped with a diode laser for 404 nm wavelength excitation and an argon laser for 488 nm wavelength excitation. Imaging was performed using 60×/1.4 NA oil immersion objective (Nikon, Japan). The three-channel RGB detector filters (band-pass filters 450/17, 545/45 and 688/67 for blue, green, and red channels, respectively) were used. Hoechst 33258 was excited at 404 nm, Au NCs was excited at 488 nm. The cells were incubated at 37 °C in the Microscope Stage Incubation System (OkoLab, Pozzuoli, Italy) in a humidified atmosphere containing 5% of CO_2_ (0.80 Nl/min O_2_ and 0.04 Nl/min CO_2_) during imaging. Image processing was performed using the Nikon EZ-C1 Bronze version 3.80 and ImageJ 1.46 software.

### 2.8. The Phototoxicity of Au NCs in Cells

To determine the phototoxicity of the Au NCs Rh+ and Au NCs Rh− in cell cultures, MCF-7 and MDA-MB-231 cells were seeded into 8-chambered cover glass plates (Nalge Nunc International, Rochester, NY, USA) with a density of 3 × 10^4^ cells/chamber and incubated at 37 °C in a humidified atmosphere containing 5 % of CO_2_ for 24 h. Subsequently, the cells were treated with 13.75 mg/mL of Au NCs Rh+ or Au NCs Rh− and then incubated under the same conditions for the next 24 h. The cells being incubated with medium alone were taken as a control. After 24 h, the initial incubation medium with Au NCs was carefully aspirated and replaced with a fresh one. Then, the cells were irradiated with a xenon light source MAX-302 (Asahi Spectra, Tokyo, Japan) through a 400/10 nm bandpass filter (power density of 30 mW/cm^2^) up to 20 J/cm^2^, 40 J/cm^2^, or 60 J/cm^2^ dose. The other group of cell samples was kept in the dark. After irradiation, the samples were put in the incubator for an additional 24 h before phototoxicity assessment. 

Two dyes were used to mark live and dead cells: green-fluorescent calcein-AM (λ_ex_ = 488 nm, Thermo Fisher) for indication of the intracellular esterase activity, and red-fluorescent propidium iodide (λ_ex_ = 543 nm, ROTH, Bavaria, Germany) for indication of the loss of plasma membrane integrity. The survival of exposed and control cells after staining with fluorescent dyes was assessed using a laser scanning confocal microscope. Imaging was performed using 20×/0.5 NA objective (Nikon, Japan). The three-channel RGB detector filters (band-pass filters 450/17, 515/15 and 604/35 for blue, green, and red channels, respectively) were used. The cells during imaging were maintained as described in Section 2.6.

The phototoxicity on both cell lines was additionally quantified by counting viable and non-viable cells from obtained confocal imaging pictures, using ImageJ software. The viability of cells was calculated by dividing the number of viable cells by the number of all the cells. The data of cell viability were shown as the mean values of viability ± standard deviation (SD). Statistical analysis was performed using the two-tailed Student’s t-test; differences were considered significant at *p* ≤ 0.05.

## 3. Results and Discussion 

### 3.1. Optimization of Au NCs Synthesis

**Figure 1:**
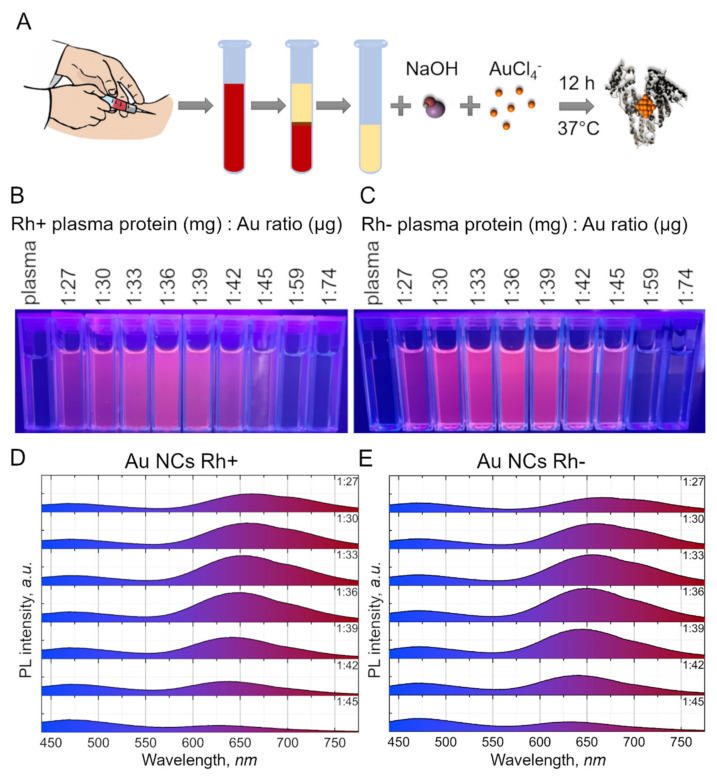
(**A**) schematic representation of Au NCs synthesis procedure with plasma proteins. Firstly, blood plasma was obtained from donors. Then, plasma was mixed with NaOH and HAuCl_4_ solutions and left for 12 h at the temperature of 37 °C under vigorous stirring. (**B**–**E**)—synthesized Au NCs photoluminescence changes due to impact of different protein—gold ratios used for Au NCs synthesis. Photoluminescence of Au NCs Rh+ (**B**) and Au NCs Rh− (**C**) colloidal solutions under UV excitation. Photoluminescence spectra (λ_ex_ = 405 nm) of Au NCs Rh+ (**D**) and Au NCs Rh− (**E**) synthesized using different protein—gold ratios. The ratios are indicated at the top right corner for each spectrum.

Schematic illustration of Au NCs synthesis and PL spectra of synthesized Au NCs Rh+/Rh− is presented in Figure 1A. The colloidal suspensions of Au NCs were stabilized with the human plasma proteins. The suspension has a light yellow-orange appearance in color at the daylight and shows a bright red PL under UV-blue radiation (Figure 1B,C). The PL spectral band maxima are around 650 nm for Au NCs synthesized in positive (Rh+) and negative (Rh−) resus factor of blood group O human plasma. No differences of PL spectra were detected between synthetized Au NCs in Rh+ or Rh− human plasma. Additionally, no variation was observed between Au NCs synthesized from plasma of different donors. We synthesized Au NCs in plasma of 6 blood donors (3 were from the group O Rh+ and 3 were from the group O Rh−) and the PL spectra of Au NCs were the same.

In order to optimize Au NCs biosynthesis, several different volumes of chloroauric acid were chosen to optimize the mass ratio of plasma proteins to gold in the solution. The PL of colloidal solutions under UV excitation demonstrated that Au NCs possessed bright PL when values range from 1 mg: 27 µg to 1 mg: 45 µg for the proteins to gold mass ratio (Figure 1). Au NCs synthesized using other ratios (lower than 1 mg: 27 µg or higher than 1 mg: 45 µg) did not exhibit any detectable PL. The most intense PL were obtained from Au NCs synthesized using 1 mg: 36 µg protein to Au mass ratio. We also observed that the intensity and maximum position of the Au NCs PL band depends on the protein to Au ratio used for synthesis. The increase in PL intensity was detected with the increasing of the protein to Au ratio from 1 mg: 27 µg till 1 mg: 36 µg and decreasing when the mass ratio was further increased to 1 mg: 45 µg. Simultaneously, the mass ratio variation caused a hypsochromic shift of the Au NCs PL band maximum from ~660 nm at mass ratio 1 mg: 27 µg to ~628 nm at mass ratio 1 mg: 45 µg (Figure 1D,E). We identified that the optimal protein to gold mass ratio for Au NCs stabilization is 1 mg: 36µg. Thus, this ratio was used for all further investigations.

### 3.2. Characterization of Synthesized Au NCs

**Figure 2:**
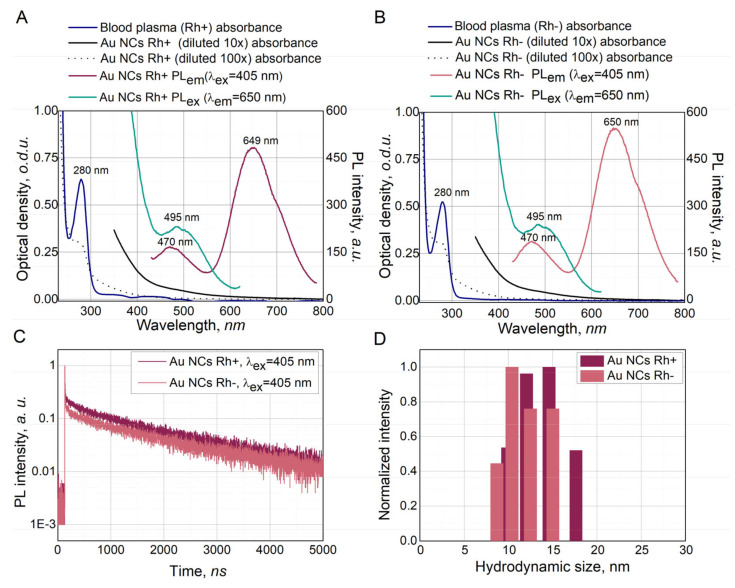
Optical properties of Au NCs Rh+ (**A**) and Au NCs Rh− (**B**): absorbance (black), photoluminescence (red), photoluminescence excitation (blue). Photoluminescence lifetimes of Au NCs Rh+ and Au NCs Rh− (**C**), hydrodynamic radius of Au NCs Rh+ and Au NCs Rh− (**D**).

More detailed optical properties of Au NCs Rh+ and Au NCs Rh− are presented in Figure 2A,B for Au NCs stabilization at the optimal plasma protein to Au mass ratio of 1 mg: 36 µg. The absorption spectra of both Au NCs Rh+ and Au NCs Rh− had a band with maximum at 280 nm, which coincides with tryptophan amino acid absorption of blood plasma band proteins. The PL spectra of Au NCs Rh+ and Au NCs Rh− are similar to other protein-stabilized Au NCs and have two PL bands at 470 nm and 650 nm wavelength. Both Au NCs Rh+ and Au NCs Rh− suspensions have lower intensity PL band at 470 nm, which is not related to the PL of Au NCs [47,48]. The more intense PL band with peak at 650 nm has slightly higher (~12%) intensity in Au NCs Rh− compared to Au NCs Rh+, but the spectral features are similar for both Au NCs synthetized in Rh+ and Rh− blood plasma. PL excitation spectra of both Au NCs Rh+ and Au NCs Rh− exhibit the peak around 495 nm. Since absorption and PL excitation do not coincide for the Au NCs, we can affirm that both, photoluminescent and non-photoluminescent, species were formed during the synthesis of Au NCs. Overall, despite slight shifts in wavelength of band maxima for absorbance, PL and PL excitation, the Au NCs Rh+ and Au NCs Rh− spectra are typical for protein stabilized Au NCs [18]. Additionally, Au NCs Rh+/Rh− solutions remained colloidally stable, and PL intensity of Au NCs remained higher that 90% of the initial PL intensity after one month (see for supporting information in Figure S1). Even after storing the suspensions for four months at 4 °C, the PL intensity remained higher than 80% of initial PL intensity of freshly synthesized solutions. This shows an excellent stability of Au NCs Rh+/Rh−.

Photoluminescence decay lifetimes were measured upon 405 nm excitation (Figure 2C). The Au NCs Rh+ and Au NCs Rh− fluorescence (PL band at 650 nm) decay was approximated by three exponential decay function with short (τ_1_ = ~5 ns), intermediate (τ_2_ = ~200 ns) and long (τ_3_ = ~1.7 µs) lifetimes (see supporting information in Figure S2 and Table S1). This result is very similar to BSA–Au NCs PL decays previously reported in the literature [49]. It was proposed that the BSA–Au NCs short PL decay component is related to prompt fluorescence of Au NCs in the albumin surrounding, whereas the long photoluminescence decay component is related to delayed fluorescence of BSA–Au NCs [50]. 

It was shown by Wong et.al. that after formation of Au NCs in BSA two oxidation states of Au is present [51]. This indicates that in such Au NCs semi ring Au and core Au structures are formed similar to presented in Zhu et al. publication [52]. It was shown by Yang et al. [53] that noble metal nanoclusters exhibit short (ns range) and long lifetimes (us range) PL lifetimes that can be assigned to metal centered emission and ligand (protein) centered emission, respectively.

Long lived PL excited state of Au NCs Rh+/Rh− makes highly favorable conditions for the generation of various radicals (ROS and singlet oxygen). 

Hydrodynamic size measurements (Figure 2D) showed that Au NCs Rh+ and Au NCs Rh− are very similar in size. Measured hydrodynamic diameter of synthesized Au NCs was around 12 nm, in both cases, and was slightly higher compared to the previously reported hydrodynamic diameter of BSA–Au NCs [7]. 

### 3.3. Singlet Oxygen and Other Reactive Oxygen Species Generation

**Figure 3:**
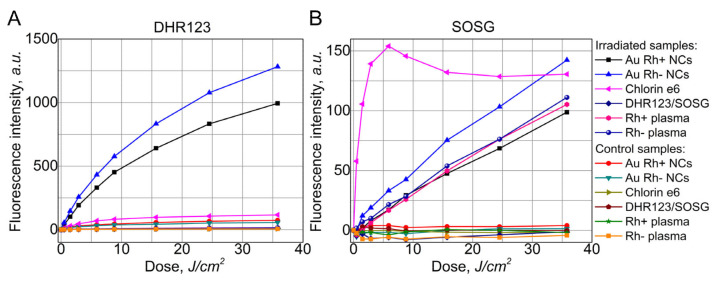
ROS generation detected with DHR123 (**A**) compared to ^1^O_2_ generation detected with SOSG (**B**). Samples were irradiated with 405 nm laser up to 35.77 J/cm^2^.

In order to evaluate Au NCs Rh+ and Au NCs Rh− capability to generate ^1^O_2_ and ROS upon light irradiation, two fluorescent sensors were chosen: SOSG for ^1^O_2_ detection and DHR123 for peroxides and peroxynitrites detection. A well-known photosensitizer, Ce_6_, was used as a positive control for the estimation of quantity of the generation of ROS. Figure 3 shows intensity of DHR132 and SOSG PL bands upon irradiation of the samples with various light doses of 405 nm wavelength light. Acquired PL spectra during irradiation are presented in supporting information Figure S3. The increase in fluorescence intensity of DHR123 was detected under irradiation with 405 nm laser in the samples with Au NCs Rh+ and Au NCs Rh−. This demonstrates the generation of ROS. On the other hand, there was no increase in the fluorescence intensity of DHR123 in non-irradiated samples. This shows that ROS were not generated without light. DHR123 fluorescence only slightly increased after irradiation with the same dose in the samples with Ce_6_ or Rh+/Rh− plasma alone (Figure 3A). The fluorescence of SOSG increased in all irradiated samples, though the rate of increment was different, which shows that generation efficacy of the ^1^O_2_ is different for different samples. In Figure 3B, it is demonstrated that Ce_6_ generates ^1^O_2_ very quickly, compared to Au NCs, however, the maximum amount of ^1^O_2_ is very similar to the amount which is generated by Au NCs after longer irradiation. Additionally, we observed that human plasma itself also generates ^1^O_2_ upon light exposure, which could be easily explained by the presence of endogenous photosensitizers, such as uroporphyrin, coproporphyrin, and protoporphyrin, in plasma [54]. 

Thus, our studies have demonstrated that Au NCs stabilized with plasma proteins can generate ROS under 405 nm light irradiation. Additionally, we identified that Au NCs Rh+ and Au NCs Rh− more efficiently generate not ^1^O_2_, as porphyrin type photosensitizers, but other ROS, as evidenced by a higher DHR123 fluorescence intensity increase.

We roughly estimated ROS and singlet oxygen generation efficiency of Au NCs by comparing it to well-known photosensitizer Ce_6_. This was completed by calculating the ratio of ROS (or ^1^O_2_) generation efficiency between investigated particles and Ce_6_ by using the equation:

(2) $$\frac{GE_{x}}{GE_{Ce6}}=\frac{\Delta I_{FL_{x}}}{\Delta I_{FL_{Ce6}}}\times \frac{1-10^{-OD_{Ce6}}}{1-10^{-OD_{x}}},$$

where GE is ROS (or ^1^O_2_) generation efficiency, $\Delta I_{FL_{Ce6}}$—increase in sensor (DHR123 or SOSG) fluorescence intensity during irradiation in Ce_6_ solution, $\Delta I_{FL_{x}}$—increase in sensor (DHR123 or SOSG) fluorescence intensity during irradiation in investigated solution, $1-10^{-OD_{Ce6}}$—fraction of absorbed light by Ce_6_ solution, $1-10^{-OD_{x}}$—fraction of absorbed light by investigated solution.

Since Ce_6_ bleaches very fast under irradiation, calculations were performed only using the first three experimental points. This was done to avoid the errors caused by changes in Ce_6_ concentration due to photobleaching. 

Calculated ROS generation ratios for Au NCs Rh+ and Au NCs Rh− were 2.7 and 2.3, respectively. This indicates that investigated Au NCs generates at least two times more ROS than Ce_6_. Plasma (Rh+, Rh−) samples did not generate significant amount of ROS (~0.01 of ROS generated by Ce_6_). However, efficiency of singlet oxygen generation by Au NCs Rh+ and Au NCs Rh− was low when compared to Ce_6_. It was 0.04 and 0.062, respectively for Au NCs Rh+ and Au NCs Rh−. These values were similar to ones calculated for plasma (Rh+, Rh−) samples. The results provide evidence that photosensitizing effect of Au NCs via ROS generation in the form of singlet oxygen is low for these nanoclusters.

### 3.4. Au NCs Accumulation in Breast Cancer Cells

**Figure 4:**
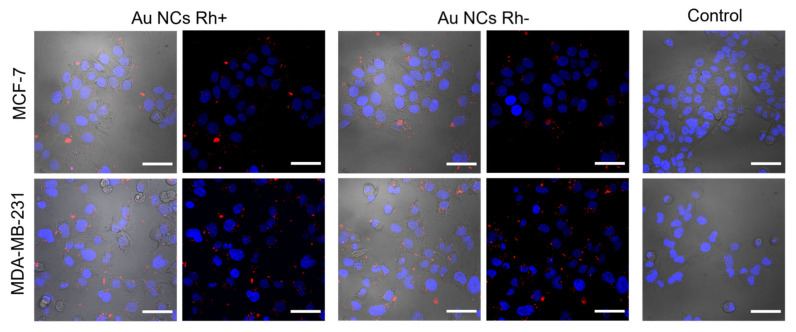
Confocal microscope images of Au NCs Rh+ and Au NCs Rh− accumulation in MCF-7 and MDA-MB-231 cancer cells. Photoluminescence of Au NCs (λ_ex_ = 488 nm) represented in red color and fluorescence of nuclei stain Hoechst (λ_ex_ = 404 nm) in blue color. Scale bars correspond to 50 µm.

In order to assure that Au NCs Rh+ and Au NCs Rh− are suitable for biological application, accumulation of these Au NCs was investigated in live breast cancer cells MCF-7 and MDA-MB-231 by imaging with laser scanning confocal microscope. Both Au NCs Rh+ and Au NCs Rh− accumulate in MCF-7 and MDA-MB-231 cells (Figure 4). The difference of the accumulation pattern was not observed between Au NCs Rh+ and Au NCs Rh− in our experiments. From combined confocal fluorescence and transmission microscopy images (Figure 4), we can state that Au NCs Rh+/Rh (red color) were clustered in the cytoplasm of the cells, often near the nuclei (blue color), but not inside the nuclei. The Au NCs Rh+/Rh were not distributed homogenously in the cells, but clustered in some compartments, probably vesicles, such as endosomes or lysosomes. Similar accumulation and localization of Au NCs was previously observed with BSA–Au NCs [55] and, generally, this type of nanoparticle internalization into the cells commonly proceeds via endocytosis [56]. Additionally, a small amount of Au NCs aggregates/agglomerates could form in the incubation medium and then enter cells via micropinocytosis mechanism, leading to appearance of bright large clusters visible inside the cells. Additionally, the Au NCs Rh+/Rh− uptake pattern was similar in both, MCF-7 and MDA-MB-231, cell lines. 

Furthermore, Au NCs Rh+/Rh− could be detected in cells by using nonlinear optical microscopy [8]. The third harmonic generation signal of Au NCs Rh+/Rh− was observed in fixed MCF-7 and MDA-MB-231 cells (Figure S4). Thus, Au NCs Rh+/Rh− has necessary properties which could be used for multimodal optical biopsy or identification of tumor boundaries during surgical operation. 

### 3.5. Biocompatibility of Au NCs

**Figure 5:**
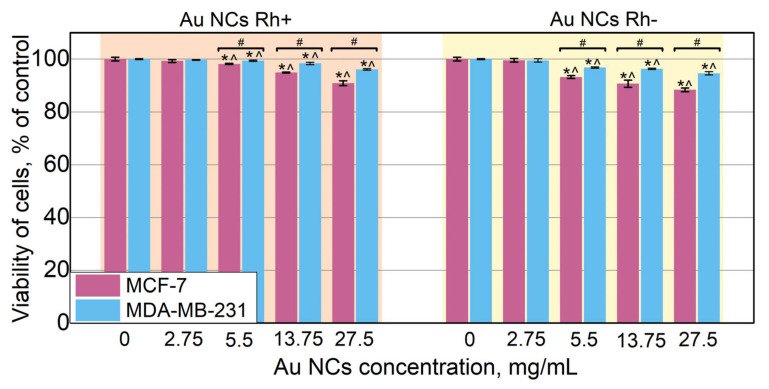
Viability of MCF-7 and MDA-MB-231 cancer cells after 24 h treatment with different concentrations Au NCs Rh+ or Au NCs Rh−. The data of cell viability were shown as the mean values of three independent experiments ± standard deviation (SD). Statistical analysis was performed using the two-tailed Student’s t-test; differences were considered significant at *p* ≤ 0.05; * *p* ≤ 0.05, # indicates significant differences between the MCF-7 and MDA-MB-231 cell lines (*p* ≤ 0.05); ^ indicates significant difference between Au NCs Rh+ and Au NCs Rh− (*p* ≤ 0.05).

Though confocal imaging did not show any obvious damage of the cells after treatment with Au NCs Rh+ and Au NCs Rh−, the cell viability was assessed after dark treatment with various concentrations of Au NCs Rh+/Rh−. For viability evaluation, the extracellular presence of LDH in cell media was detected by using commercial LDH Cytotoxicity Assay kit (Thermo Fisher, USA). The viability was calculated as a percentage of surviving cells to control cells without Au NCs treatment. As it is shown in Figure 5, there was no effect on cell viability after cells were incubated with 2.75 mg/mL of Au NCs Rh+ or Au NCs Rh−. Only a slight decrease in cell viability was observed after incubation with higher concentrations, though MCF-7 cells were more affected compared to MDA-MB-231 cells. However, even after treatment with 27.5 mg/mL Au NCs, viability of cells remains at around 90%, which allows to conclude, that both Au NCs Rh+ and Au NCs Rh− are non-toxic for MCF-7 and MDA-MB-231 cells under the dark treatment conditions. Similar cytotoxicity was observed by Ungor et al. [57] for dark treatment of human lymphocytes with HSA stabilized Au NCs. The same LDH release assay was employed for the viability assessment. The treatment resulted in only a 5% decrease in COLO-720L cell viability after incubation with 25, 50, and 75 mg/L HSA–Au NCs solutions. In addition, there was no cytotoxicity observed for HUT-78 cells after the same treatment [57]. On the other hand, higher cytotoxicity was observed when, for example, 50 µM of HSA–Au solution was used, decreasing the viability of MDA-MB-231 cells by 50% [27]. Li et al. investigated cytotoxicity of Au NCs stabilized with proteins from egg white and fetal bovine serum using WST-1 assays and did not observe any harmful effect [34]. Most often investigated BSA–Au NCs are also considered to be non-toxic [57,58,59]. However, few authors, including our group, have demonstrated that BSA–Au NCs can induce ROS generation even without light exposure and, therefore, slightly affect viability of cells [7,55,60,61,62]. These differences may occur due to use of different viability assay protocols and points to the need for more comparative studies of cell viability. Previously mentioned Ungor et at. measured viability of cells after treatment with protein stabilized Au NCs using few methods and showed that in some cases results between different methods varies [57]. From our experience, Au NCs stabilized with human plasma proteins, as presented in this article, are more biocompatible compared with BSA–Au NCs. However, in this study, the buffer exchange procedure was performed after Au NCs synthesis, while it was not implemented in our previous studies of BSA–Au NCs. We believe this additional step could be crucial for cell viability studies. 

**Figure 6:**
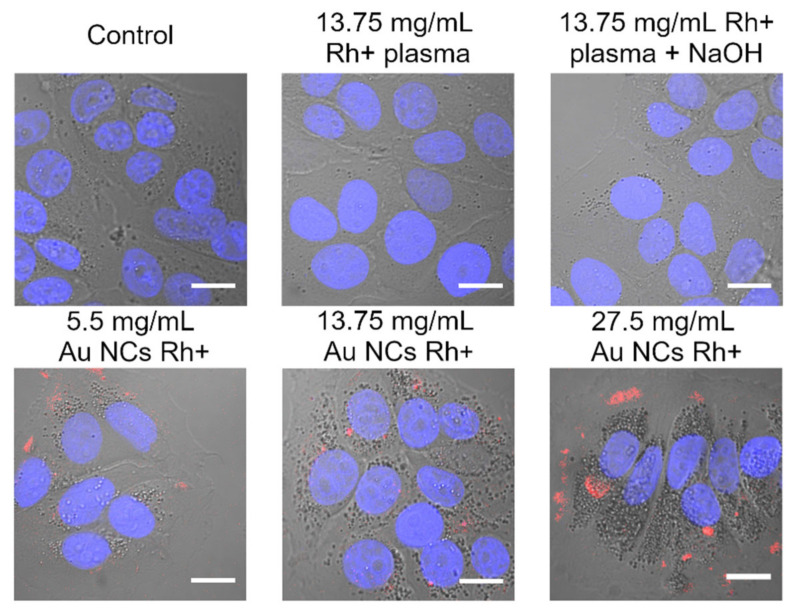
Morphological changes of MCF-7 cancer cells after treatment with different concentration of Au NCs Rh+ (bottom row) compared to controls (top row). Photoluminescence of Au NCs (λ_ex_ = 488 nm) represented in red color and fluorescence of nuclei stain Hoechst (λ_ex_ = 404 nm) in blue color. Scale bars in all images correspond to 15 µm.

Although viability of cells remains high and there is no obvious damage to the cells, some morphological changes were observed after incubation with Au NCs. In Figure 6, the confocal microscope images of MCF-7 control, and treated cells are demonstrated. As it is seen, the cells in all images have easily noticeable vesicles, but the number of vesicles differs depending on the incubation conditions. Control cells and cells incubated with only Rh+ plasma or Rh+ plasma with NaOH (which is used during Au NCs synthesis), have a small amount of the vesicles, and morphologically look the same as the control. Addition of Au NCs Rh+ increases the number of visible vesicles in cells and the increase correlates with added amount of Au NCs. Cells treated with 5.5 mg/mL of Au NCs Rh+ have only a slight increment of visible vesicles compared to the control cells, whereas after incubation with 13.75 mg/mL or 27.5 mg/mL Au NC Rh+ much more vesicles appear. Additionally, PL of Au NCs Rh+ do not necessarily overlap with those vesicles. All previously described effects also were seen in MDA-MB-231 cells and both MCF-7 and MDA-MB-231 cells incubated with Au NCs Rh−(data not shown). Interestingly, the same effect for MCF-7 and MDA-MB-231 cells morphology was also observed after treatment with BSA–Au NCs [55]. Thus, this effect probably is Au NCs related, independently from proteins used for Au NCs stabilization. We think that the increase in vesicles in cells is not specific to Au NCs and might be linked to the overall NPs impact on the cells. Unfortunately, morphological changes of cells after incubation with NPs are rarely addressed in the publications. Usually, appearance of such structures is attributed to accumulation of NPs, especially when the uptake of metallic or magnetic NPs are investigated, however, our results do not show colocalization of vesicles and PL of Au NCs. One of the reasons could be that NCs aggregate inside cellular vesicles or due to interaction with cellular proteases, changed pH values lost PL, thus could not be detected with microscope. On the other hand, endosomes with and without NPs are formed, during incubation with NPs [63]. Thus, if our discussed vesicles are endosomes, not all of them may contain Au NCs; some endosomes may form as a result of the cell’s natural metabolic activity. Ma et al. demonstrated the size dependent cells morphology changes after incubation with 10, 25, and 50 nm Au NPs: Au NPs interferes with lysosomal functions which cause enlargement of the lysosomes [64]. Returning to our results, our hypothesis is that treatment with Au NCs induces activation of metabolism in cells, which leads to an increased number of vesicles (e.g., endosomes) or contrary, Au NCs disrupt some metabolic processes, which also causes an increase in the number of vesicles (e.g., enlarged lysosomes) in the cells.

### 3.6. Photodynamic Effect

**Figure 7:**
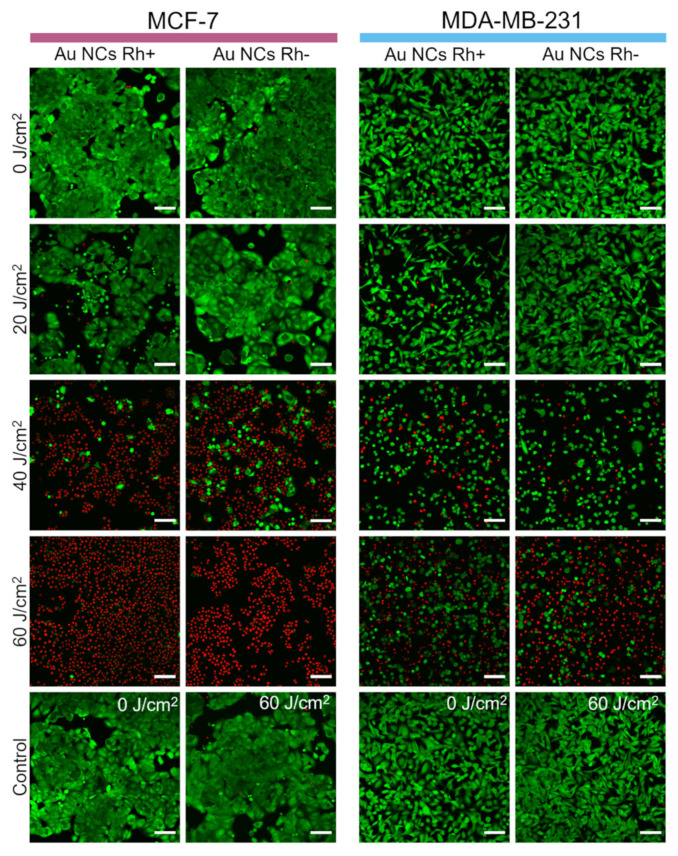
Au NCs Rh+ and Au NC Rh− photodynamic effect for MCF-7 and MDA-MB-231 cells, after irradiation with 400/10 nm light. The cells were stained with fluorescent viability dyes: calcein AM (green) was used for live cells and propidium iodide (red) for dead cells visualization. Scale bars in all images correspond to 100 µm.

In order to evaluate the photodynamic effect of Au NCs in the cells, MCF-7 and MDA-MB-231 cells were preincubated with Au NCs Rh+ or Au NCs Rh− and then irradiated with blue light (400/10 nm bandpass filter, power density of 30 mW/cm^2^). We have chosen to use 400/10 nm light for irradiation, because PL excitation of such wavelength is more effective and the light penetration in cell monolayers is unrestricted, however, for in vivo applications, longer waves of light should be chosen for larger penetration depth, which would not be a problem since Au NCs Rh+/Rh− have a broad PL excitation spectrum (Figure 2A,B). Different irradiation doses were tested to identify optimal irradiation conditions, as well as to determine if the photodynamic effect is dose dependent. After irradiation the cells were stained with fluorescent viability dyes calcein AM, which stains live cells, and propidium iodide, which stains nuclei of dead cells. After the 20 J/cm^2^ irradiation dose, MCF-7 and MDA-MB-231 cells remained viable (green color, Figure 7), however, the morphology of some cells changed: they partially detached from the microscopy plate surface and the shape of cells became round or apoptotic (supporting information Figure S5). After 40 J/cm^2^ irradiation, the viability of MCF-7 cells drastically changed. Most of the cells were non-viable (red color, Figure 7). On the other hand, most of MDA-MB-231 cells remained viable, though almost all viable cells had round or apoptotic morphology. As shown in Figure 7, 100 % of MCF-7 cells were non-viable after a 60 J/cm^2^ irradiation dose, while only approximately half of MDA-MB-231 cells were non-viable, but another half was also affected, and their shape became round or apoptotic. Irradiation with both Au NCs Rh+ and Au NCs Rh− present, had similar effects on the cells.

**Figure 8:**
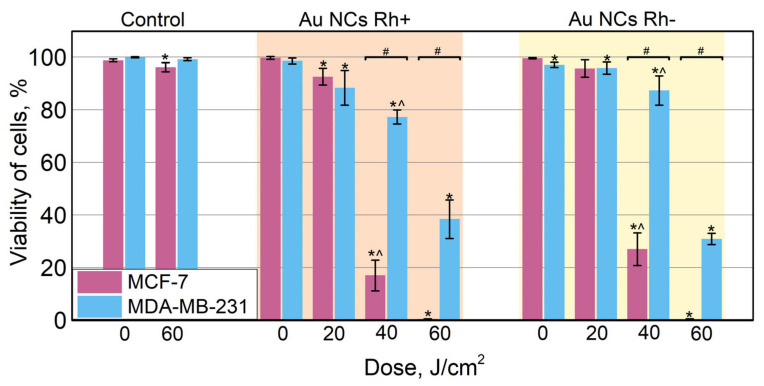
Quantitative evaluation of Au NCs Rh+ and Au NC Rh− photodynamic effect for MCF-7 and MDA-MB-231 cells, after irradiation with 400/10 nm light. Error bars show standard deviations. Statistically significant differences compared to the control cells are shown (*, *p* ≤ 0.05); # indicates significant differences between the MCF-7 and MDA-MB-231 cell lines (*p* ≤ 0.05); ^ indicates significant difference between Au NCs Rh+ and Au NCs Rh− (*p* ≤ 0.05).

Additionally, the quantitative evaluation of the photodynamic effect in MCF-7 and MDA-MB-231 cells is presented in Figure 8. Viability of cells without nanoclusters after irradiation with 60 J/cm^2^ dose remained unaffected with calculated viability of both MCF-7 and MDA-MB-231 cells higher than 96%. The same case is for the cells which were incubated with Au NCs but were not irradiated with 405 nm light. The viability of all those samples was 97% or higher. After irradiation with a 20 J/cm^2^ dose, the viability of cells, pre-incubated with Au NCs slightly decreases. The calculated viability of cells pre-incubated with Au NCs Rh+ was a little bit lower (MCF-7—93%, MDA-MB-231—88%), compared to the cells pre-incubated with Au NCs Rh− (both 96%). However, statistical analysis did not show any statistically significant differences between the viability of cells pre-incubated with Au NCs Rh+ or Au NCs Rh−. On the other hand, differences appear after a higher irradiation dose. First, both Au NCs Rh+ and Au NCs Rh− had a higher phototoxic effect for MCF-7 cells than for MDA-MB-231 cells. This result corresponds with our previous study, where BSA–Au NCs had higher phototoxicity for MCF-7 cells compared to MDA-MB-231 cells [7]. The viability of MCF-7 cells after irradiation with 40 J/cm^2^ dose decreased to 17% and 27% when pre-incubated with Au NCs Rh+ and Au NCs Rh−, respectively. After 60 J/cm^2^ exposure, all MCF-7 cells were dead, as seen in both Figure 7 and Figure 8. Meanwhile, the viability of MDA-MB-231 cells after irradiation with the 40 J/cm^2^ dose remained 77% and 87% when pre-incubated with Au NCs Rh+ and Au NCs Rh−, respectively. After a higher dose (60 J/cm^2^) 38% (Au NCs Rh+) and 31% (Au NCs Rh−) of MDA-MB-231 cells survived. Another statistically significant difference between Au NCs Rh+ and Au NCs Rh− was observed: Au NCs Rh+ were more phototoxic compared to Au NCs Rh−. On the other hand, slight differences were noticed also assessing dark toxicity (Figure 5), where the viability of cells incubated with Au NCs Rh− was lower compared to incubated with Au NCs Rh+. 

There are only few papers where the phototoxicity of Au NCs was investigated; however, due to variation of the use of Au NCs, differences in applied methods or insufficient information about irradiation doses and used light power densities, it is difficult to objectively compare the obtained results. 

## 4. Conclusions

In this article, we have demonstrated that it is possible to synthesize the Au NCs directly in patients’ blood plasma, and we realized the personalized diagnostics and treatment simultaneously by using the theranostic agent of Au NCs Rh+/Rh−. Au NCs synthesized from the plasma of six independent donors (three of blood group O Rh+ and three of blood group O Rh− plasma) showed the reproducible results. Thus, the suggested synthesis method is universal, because the same Au NCs is synthesized regardless of Rh factor or plasma donor. Our study shows that human plasma proteins stabilized Au NCs accumulate in different tumorigenicity human breast cancer cells, MCF-7 and MDA-MB-231, and do not cause cytotoxicity in the dark. Hence, these Au NCs are biocompatible. On the other hand, plasma proteins stabilized Au NCs after irradiation with 400/10 nm light generate significant amount of ROS and induce photodynamic effect in the cells, which is dose dependent. Because of light dependent phototoxicity, these Au NCs could be used simultaneously as agents for optical biopsy and as photosensitizers in photodynamic tumor therapy. Overall, our results demonstrate that human plasma proteins stabilized Au NCs could be applied for personalized cancer theranostics. Finally, we agree with the statement of Cifuentes-Rius et al. that a bright future awaits for Au NCs in cancer theranostics [9], though a lot of research still has to be completed.

## Data Availability

The data presented in this study are available in this article and supplementary material.

## Supplementary Materials

The following supporting information can be downloaded at: https://www.mdpi.com/article/10.3390/cancers14081887/s1, Figure S1: Photoluminescence spectra of Au NCs Rh+ and Au NCs Rh− measured at different time after synthesis; Figure S2: Photoluminescence decay kinetics of Au NCs Rh+ and Au NCs Rh−; Table S1: Coefficients of the three-exponential fit for the photoluminescence decay curves corresponding to Au NCs Rh+ and Au NCs Rh−; Figure S3: PL spectra of Au NCs Rh+, Au NCs Rh− and Ce_6_ with ROS sensor DHR123 and PL spectra of Au NCs Rh+, Au NCs Rh− and Ce_6_ with SOSG sensor after irradiation with various doses of 405 nm light; Figure S4: Nonlinear microscopy images of fixed MCF-7 and MDA-MB-231 cells, incubated with Au NCs Rh+/Rh−; Figure S5: Au NCs Rh+ photodynamic effect for MCF-7 and MDA-MB-231 cells, after irradiation with 400/10 nm light. Reference [65] is cited in the supplementary materials.
