# Supplementary material for: Blood Plasma Stabilized Gold Nanoclusters for Personalized Tumor Theranostics
Source: Cancers (Basel). 2022 Apr 8;14(8):1887. doi: 10.3390/cancers14081887 (PMC9030650; doi:10.3390/cancers14081887)
Supplement: Supplementary file 1 [file cancers-14-01887-s001.zip › cancers-1654952-supplementary.pdf]

Supporting Information

Blood plasma stabilized gold nanoclusters for personalized tumor theranostics

Greta Jarockyte[1,2,3], Vilius Poderys[1], Virginijus Barzda[3,4,5], Vitalijus Karabanovas[1,3,6], Ričardas Rotomskis[1,3]

Contents

1. Colloidal stability 2
2. Photoluminescence decay kinetics 2
3. Detection of ROS and singlet oxygen 4
4. Nonlinear imaging 4
5. Cells′ morphology after irradiation 6
6. References 6

1. Colloidal stability

Photoluminescence spectra of Au NCs Rh+/Rh- solutions during the first 30 days after synthesis is showed in Figure S1. Au NCs Rh+/Rh- solutions remained colloidally stable and photoluminescence intensity of Au NCs remained higher that 90% of initial photoluminescence.

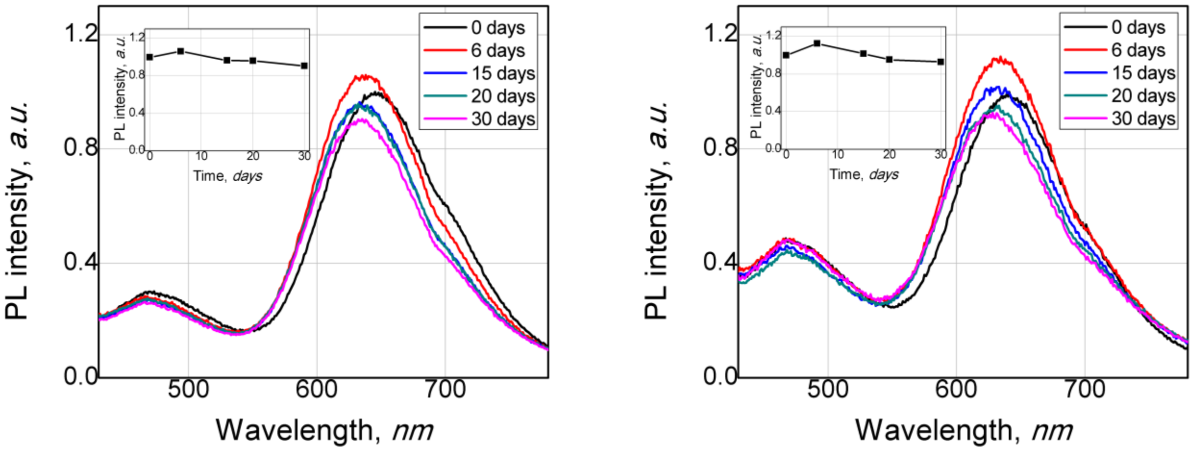

Figure S1. Photoluminescence spectra of Au NCs Rh+ (left) and Au NCs Rh- (right) measured at different time after synthesis. Inserts show dependance of PL band maximum intensity on time after synthesis. PL intensity was normalized relative to Au NCs Rh+/- band intensity measured right after the synthesis.

2. Photoluminescence decay kinetics

Photoluminescence lifetimes were registered using FLS 920 spectrometer (Edinburgh instruments Ltd., Scotland). Diode laser (λ=405 nm wavelength, pulse length - <200ps) was used for excitation of the samples. Pulse repetition rate for PL decay of the band at 650 nm was 100 kHz, for the band at 470 nm – 2 Mhz. PL decay was measured at the peak emission wavelengths of Au NCs Rh+/Rh- photoluminescence bands 650 nm (**Error! Reference source not found.** A,B) and 470 nm (**Error! Reference source not found.** C,D).

The experimental data were fitted using F900 software (Edinburgh instruments Ltd., Scotland), 3-term exponential decay fit model (1) was used to analyze the data. Data fitting using 2-term exponential model did not yield good results (χ^2> 1.2). PL lifetimes of fitted data are provided in table S1.

$$R(t) = B_1 e^{-\frac{t}{\tau_1}} + B_2 e^{-\frac{t}{\tau_2}} + B_3 e^{-\frac{t}{\tau_3}} + A \quad (1)$$

where τ_i – PL lifetimes, B_i – pre-exponential factors, and A – background.

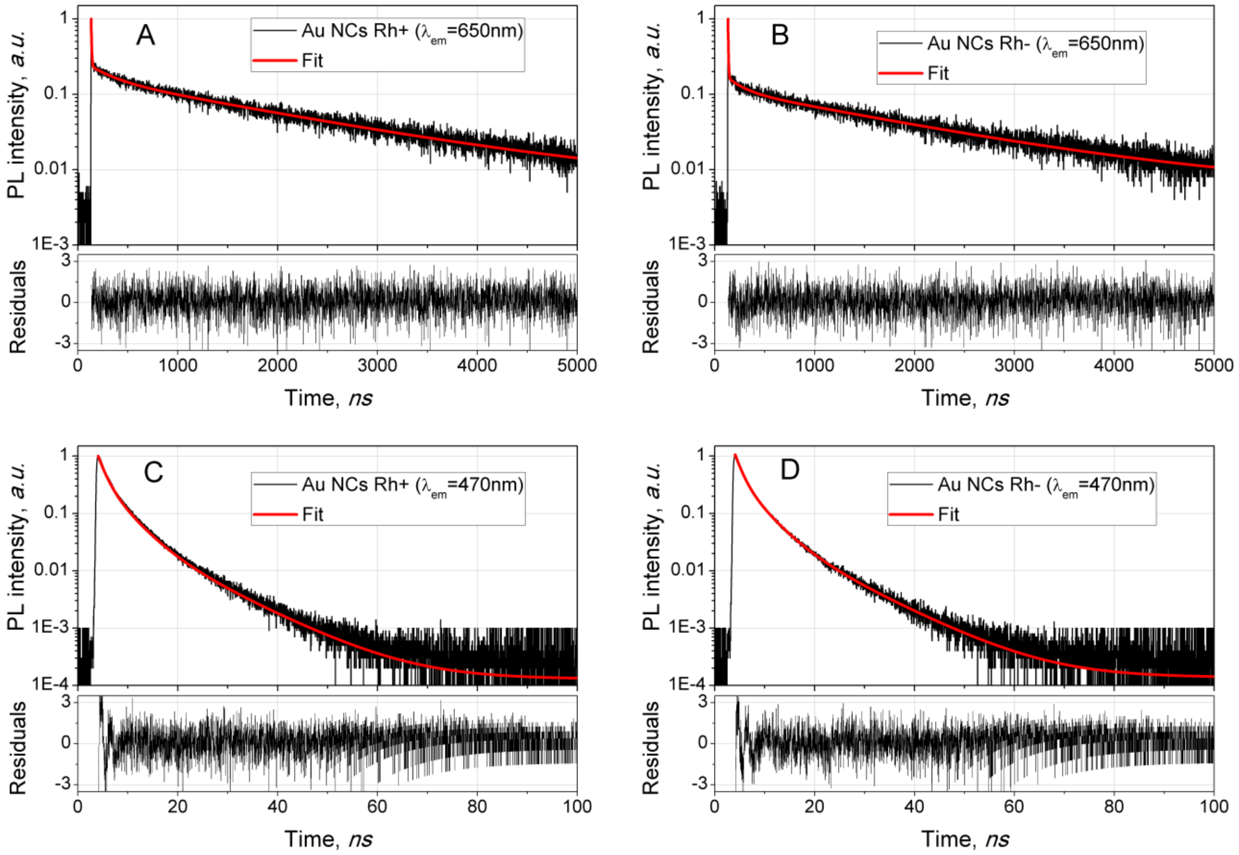

Figure S2. Photoluminescence decay kinetics of Au NCs Rh+ (**A**,**C**) and Au NCs Rh- (**B**,**D**) at λ_{em} = 650 nm (**A**,**B**) and λ_{em} = 470 nm (**C**,**D**).

Table S1. Coefficients of the three-exponential fit for the photoluminescence decay curves corresponding to Au NCs Rh+ and Au NCs Rh-.

**Au NCs Rh+**						**Au NCs Rh-**					
**λ_{em} = 650nm**			**λ_{em} = 470nm**			**λ_{em} = 650nm**			**λ_{em} = 470nm**		
τ, ns	B	Rel. %	τ, ns	B	Rel. %	τ, ns	B	Rel. %	τ, ns	B	Rel. %
4.9	0.589	1.0	1.4	0.647	29.5	3.8	0.685	1.4	1.3	0.634	28.3
233.7	0.077	6.4	3.9	0.381	49.8	156.3	0.054	4.5	3.9	0.374	49.7
1702.0	0.152	92.6	10.2	0.060	20.7	1640.0	0.107	94.1	10.2	0.063	22.0
χ^2 = 1.024			χ^2 = 1.182			χ^2 = 1.034			χ^2 = 1.169		

3. Detection of ROS and singlet oxygen

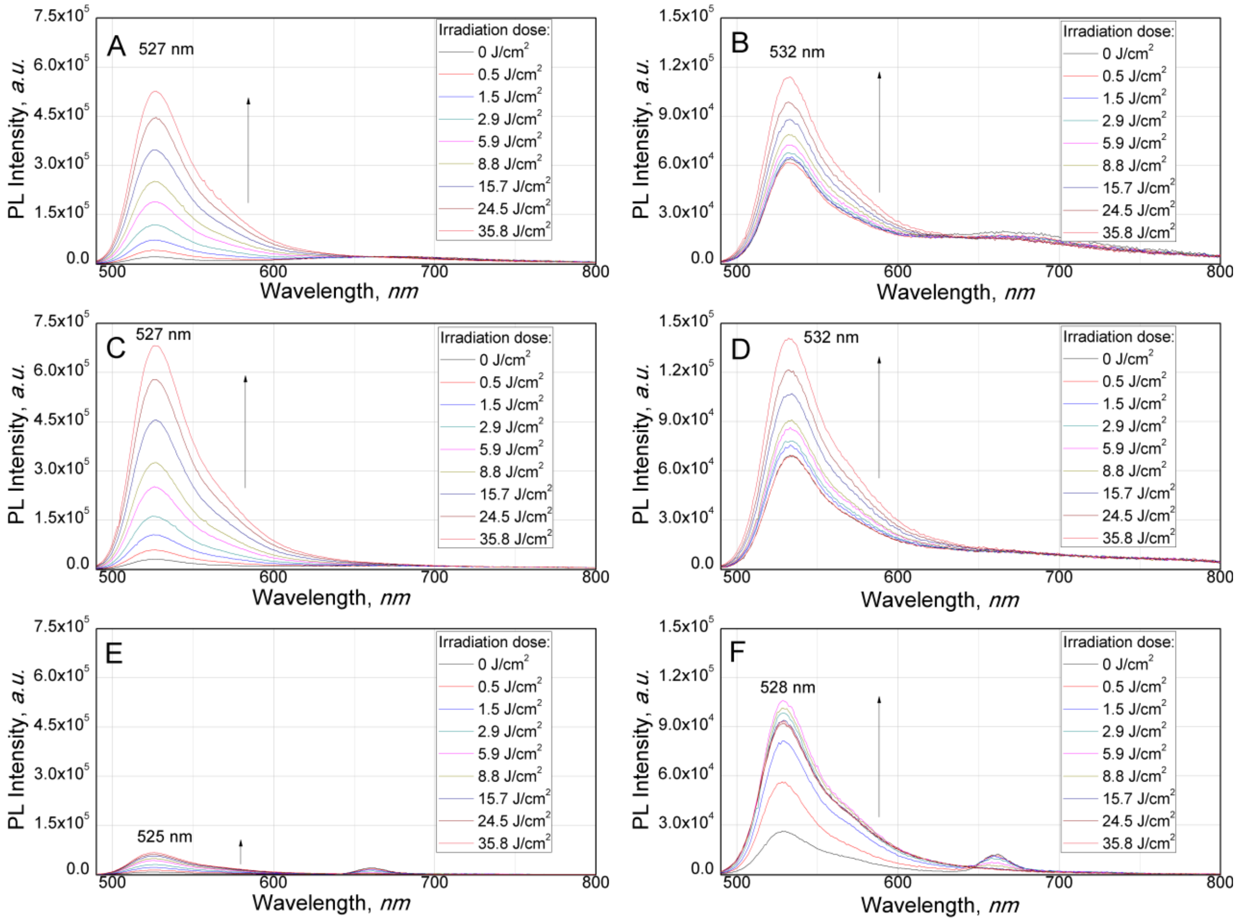

Figure S3. PL spectra of Au NCs Rh+ (**A**), Au NCs Rh- (**C**) and Ce_6 (**E**) with ROS sensor DHR123 and PL spectra of Au NCs Rh+ (**B**), Au NCs Rh- (**D**) and Ce_6 (**F**) with SOSG sensor after irradiation with various doses of 405 nm light. The doses are indicated in the figure insert.

4. Nonlinear imaging

Third-harmonic generation (THG) and multiphoton excitation fluorescence (MPEF) imaging was performed using a home built nonlinear laser scanning microscope equipped with a pulsed Yb:KGW laser (FLINT, Light Conversion, Lithuania) emitting ~ 100 fs duration pulses at 1030 nm wavelength with a 76 MHz pulse repetition rate. Galvanometric mirrors (Cambridge Technology, USA) were used for scanning. Laser light was focused on the sample with a 20× 0.75 NA objective lens (CFI Plan Apochromat, Nikon, Japan) and collected with a 0.45 NA singlet lens. Emitted light was detected with a pair of photomultiplier tubes (H10682-210, Hamamatsu, Japan) operating in photon counting mode. FBH343-10 (Thorlabs. USA) and BP460-60 (Thorlabs, USA) filters were used to filter emitted THG and MPEF, respectively. Images were acquired with ~ 23 μs pixel dwell time and 0.25 μm/px pixel size, with 20 mW laser power on the sample. Data acquisition was performed using a PCIe-6353 DAQ card (National Instruments, USA). The microscope detection and control was provided by a custom software written in LABView (National Instruments, USA).

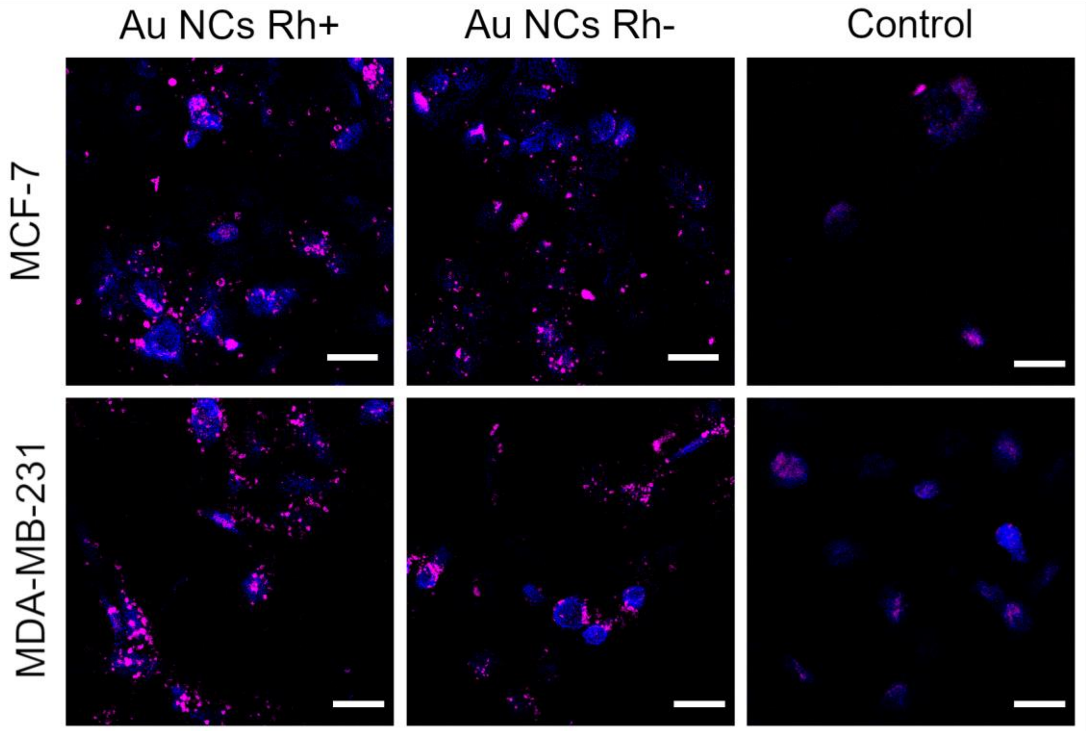

Figure S4. Nonlinear microscopy images of fixed MCF-7 and MDA-MB-231 cells, incubated with Au NCs Rh+/Rh-. Samples were excited with 1030 nm laser in order to register multiphoton excitation fluorescence of Hoechst 33258 (blue) and third harmonic generation signal generated by Au NCs Rh+/Rh- and other cellular organelles (magenta). Scale bars correspond to 20 μm.

For nonlinear microscopy imaging cells were seeded into 8-well chamber slide with removable wells (Lab-Tek, Nunc, Thermo Fisher, Denmark) with a density of $3 \cdot 10^4$ cells per chamber and maintained at 37 °C in a humidified atmosphere containing 5% of CO_2 for 24 hours. Then cells were treated with 13.75 mg/mL Au NCs Rh+/Rh- for 24 h. After incubation nuclei of cells were stained with 10 μg/mL of Hoechst 33258 (Sigma-Aldrich, Germany). Then cells were fixed with 4 % paraformaldehyde solution. Finally, fixed samples of MCF-7 and MDA-MB-231 cells with Au NCs Rh+/Rh- were visualized using nonlinear microscopy (**Error! Reference source not found.**). Previously, the third harmonic signals were obtained from the gold nanoparticles [1], thus we imaged Au NCs Rh+/Rh- treated cells with THG and MPEF microscope. As demonstrated **Error! Reference source not found.**, Au NCs Rh+/Rh- generates third harmonic signal upon 1030 nm excitation. The cell nuclei were visualized with nuclei stain Hoechst using multiphoton excitation fluorescence (λ_{ex}=1030 nm, λ_{em}=430-490 nm). The pattern of Au NCs Rh+/Rh- signal coincides with the confocal microscope images, however some third harmonic signal overlaps with fluorescence of Hoechst, indicating that Au NCs Rh+/Rh- above/bellow nuclei are detected or some nuclear structures are also visualized with THG. The third harmonic signal also appears in the control samples, without Au NCs Rh+/Rh-, showing that some cellular structures, generate third harmonic signal. Nevertheless, third harmonic signal from samples with Au NCs Rh+/Rh- are 3-5 times more intense compared with third harmonic signal from control samples, which indicates that Au NCs Rh+/Rh- are strong harmonophores. Thus the capability to visualize Au NCs Rh+/Rh- using nonlinear microscopy expands the possibilities of nanoparticle applications in photomedicine.

5. Cells' morphology after irradiation

MCF-7 and MDA-MB-231 cells were preincubated with Au NCs Rh+ or Au NCs Rh- and then irradiated with blue light (400 / 10 nm bandpass filter, power density of 30 mW/cm^2). After irradiation, cells were stained with fluorescent viability dyes calcein AM, which stain live cells, and propidium iodide, which stains nuclei of dead cells. Even after lowest dose, 20 J/cm^2, the morphology of some cells has changed. As it is seen in **Error! Reference source not found.**, some cells display blebbing, and undergoing apoptosis can be seen [2]. After 40 J/cm^2 irradiation, majority of MCF-7 cells appear already inactive, but majority of MDA-MB-231 are still alive (green color appearance), though, the morphology has changed: almost all cells became round, and some of them appear apoptotic.

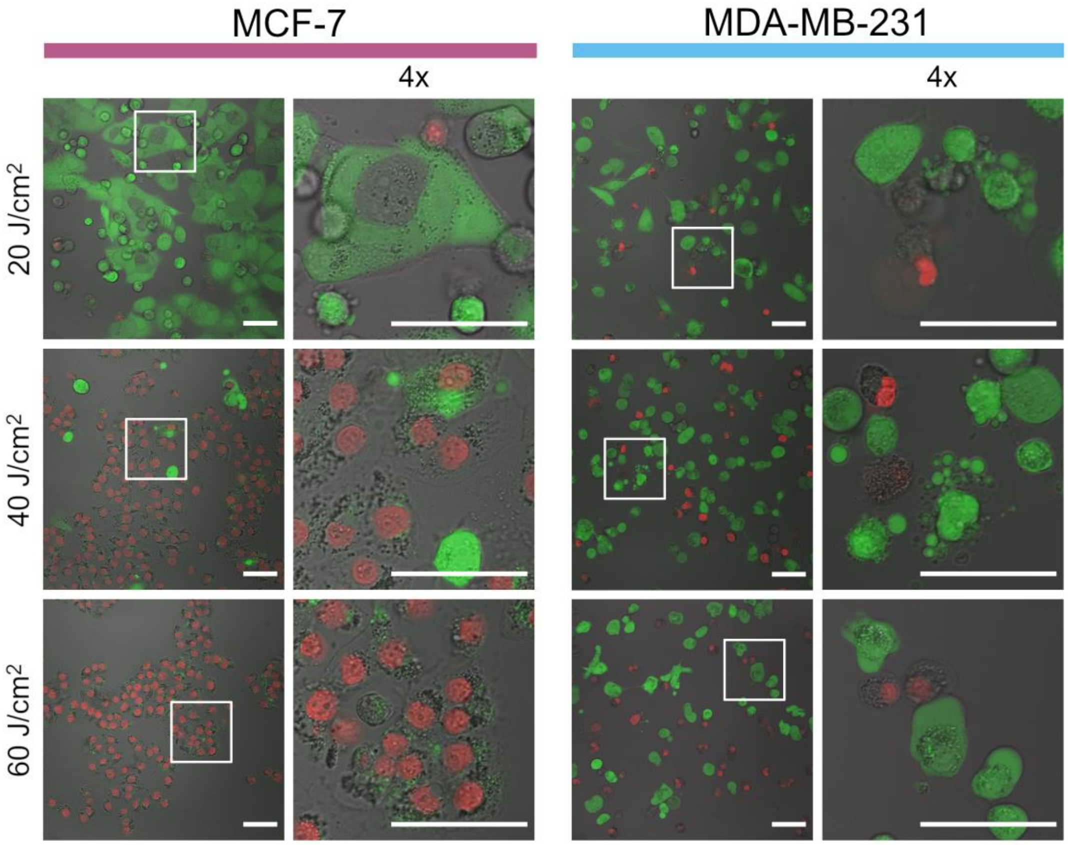

Figure S5. Au NCs Rh+ photodynamic effect for MCF-7 and MDA-MB-231 cells, after irradiation with 400/10 nm light. Cells were stained with fluorescent viability dyes: calcein AM (green) was used for visualization of live cells, and propidium iodide (red) was used for the dead cell visualization. Scale bars in all images correspond to 50 µm.

6. References

1. Lippitz, M.; van Dijk, M.A.; Orrit, M. Third-Harmonic Generation from Single Gold Nanoparticles. *Nano Lett.* **2005**, *5*, 799–802. https://doi.org/10.1021/nl0502571.
2. Van Cruchten, S.; Van den Broeck, W. Morphological and Biochemical Aspects of Apoptosis, Oncosis and Necrosis. *Anat. Histol. Embryol.* **2002**, *31*, 214–223. https://doi.org/10.1046/j.1439-0264.2002.00398.x
